# Stroma remodeling and reduced cell division define durable response to PD-1 blockade in melanoma

**DOI:** 10.1038/s41467-020-14632-2

**Published:** 2020-02-12

**Authors:** Elena Galvani, Piyushkumar A. Mundra, Sara Valpione, Pablo Garcia-Martinez, Matthew Smith, Jonathan Greenall, Rohit Thakur, Beth Helmink, Miles C. Andrews, Louis Boon, Christopher Chester, Gabriela Gremel, Kate Hogan, Amit Mandal, Kang Zeng, Antonia Banyard, Garry Ashton, Martin Cook, Paul Lorigan, Jennifer A. Wargo, Nathalie Dhomen, Richard Marais

**Affiliations:** 1 Molecular Oncology Group, Cancer Research UK Manchester Institute, The University of Manchester, Alderley Park, Manchester, UK; 2 The Christie NHS Foundation Trust, Manchester, UK; 3 Department of Surgical Oncology, The University of Texas MD Anderson Cancer Center, Houston, TX USA; 4 Olivia Newton-John Cancer Research Institute, Heidelberg, VIC Australia; 5 La Trobe University School of Cancer Medicine, Heidelberg, VIC Australia; 6 Bioceros B.V, Utrecht, The Netherlands; 7 Imaging and Cytometry, Cancer Research UK Manchester Institute, The University of Manchester, Alderley Park, Manchester, UK; 8 Histology, Cancer Research UK Manchester Institute, The University of Manchester, Alderley Park, Manchester, UK; 9 The University of Manchester, Oxford Road, Manchester, UK; 10 Department of Genomic Medicine, The University of Texas MD Anderson Cancer Center, Houston, TX USA

**Keywords:** Cancer models, Cancer immunotherapy

## Abstract

Although immune checkpoint inhibitors (ICIs) have achieved unprecedented results in melanoma, the biological features of the durable responses initiated by these drugs remain unknown. Here we show the genetic and phenotypic changes induced by treatment with programmed cell death-1 (PD-1) blockade in a genetically engineered mouse model of melanoma driven by oncogenic BRAF. In this controlled system anti-PD-1 treatment yields responses in ~35% of the tumors, and prolongs survival in ~27% of the animals. We identify increased stroma remodeling and reduced expression of proliferation markers as features associated with prolonged response. These traits are corroborated in two independent early on-treatment anti-PD-1 melanoma patient cohorts. These insights into the biological responses of tumors to ICI provide a strategy for identification of durable response early during the course of treatment and could improve patient stratification for checkpoint inhibitory drugs.

## Abstract (web summary)

Identification of clinical relevant biomarkers to predict response to immune checkpoint blockade (ICB) in melanoma remains challenging. Here, the authors show that stroma remodelling and reduced cell division are associated with durable response to anti-PD1 in a mouse model of melanoma and in ICB-treated patients.

## Introduction

Monoclonal antibodies targeting inhibitory immune checkpoints have significantly improved outcomes for advanced-stage melanoma patients^1,2^. While attention has focused on the identification of predictive biomarkers, or new treatment schedules and combinations to boost the efficacy of immune checkpoint inhibitors (ICI)^3,4^, we still do not fully understand how these drugs work, or their biological impact on cancer cells and the tumor microenvironment (TME).

Patient-derived materials, including tumor biopsies, blood, and plasma have been key to examine the dynamic interactions between the tumor and the immune system and how these influence response to ICIs^5–10^. However, the understanding of this interplay is hampered by numerous variables within humans that impact response, from the metastatic site of the lesion to the patient’s microbiome, genetic background, lifestyle, and prior lines of treatment^5–10^. While patient-derived cancer models such as xenografts and organoid cultures are tractable and easy to manipulate, they lack the native TME that plays crucial roles in dictating response to therapy and modulating immune cell function^11^. Conversely, transplantable syngeneic mouse models suffer from low intratumor heterogeneity and lack the co-evolved inflammatory environment that characterizes human melanoma. Given the complexity that each component (i.e., tumor, immune system and TME) adds to the system, models that present all the features of spontaneously developed tumors are needed to study the biological features that define response to ICIs.

We reasoned that because genetic and environmental variables are easily controlled in our BRAF^V600E^/UVR mouse melanoma model^12–14^, we would detect subtle changes in tumor and TME responses in mice receiving immunotherapy, potentially providing better understanding of the human disease. We previously reported that our preclinical model recapitulates the cardinal genomic features of human melanoma including a UVR-induced signature 7, high C-to-T mutation load, and recurrent mutations in the same top ten genes as occur in human UVR-driven melanoma^12–14^. This model retains the key features of the native immune system and TME, while additionally allowing control of the genomic and environmental variables that cannot be controlled when working with human-derived samples. Here we use this model to investigate the biological features of durable response to PD-1 blockade. We reveal that stroma remodeling and tumor proliferation are key biological processes affected by this treatment, and strikingly, we observe similar responses in two independent early on-treatment ICI cohorts of patients. Thus, the features of lasting response derived from our mouse model provide insight into human responses that are otherwise difficult to identify due to the complex nature of human populations.

## Results

### Anti-PD-1 elicits durable responses in murine melanomas

**Fig. 1:**
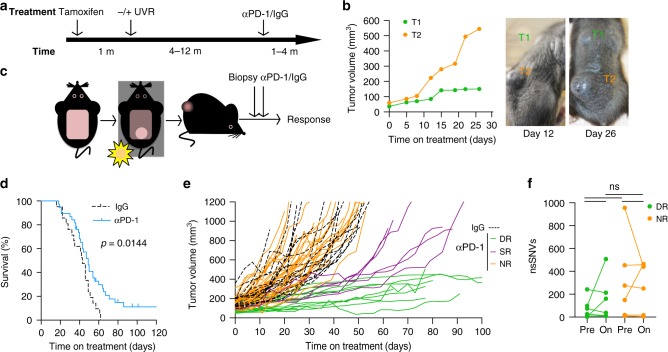
Anti-PD-1 therapy elicits durable responses in BRAF-driven murine melanoma. **a** Schematic representation of the timeline relative to our mouse melanoma model. Melanoma developed in mice a few months after the induction of oncogenic BRAF^V600E^ and exposure to ultraviolet radiation (UVR). When tumors reached ~100 mm^3^, mice were randomized to receive anti-PD-1 antibody or IgG. m, months. **b** Growth curves and photographs at indicated treatment times of a representative mouse exposed to UVR and bearing two tumors (T1 and T2) with differential response to PD-1 blockade. **c** Cartoon depicting our refined experimental approach. UVR exposure was limited to ~1 cm^2^, and a pre-treatment biopsy was collected from established tumors (~100 mm^3^). **d** Kaplan–Meier curves showing survival of mice bearing single tumors treated with anti-PD-1 (*n* = 38 animals) or IgG (*n* = 21 animals). Two-sided log-rank test was performed to determine statistical difference between the survival curves. **e** Tumor growth curves from animals treated with anti-PD-1 (continuous green/orange/purple lines, *n* = 53 tumors) or IgG (dashed black lines, *n* = 19 tumors) defines three cohorts: DR durable response (green, *n* = 13 tumors), SR short-lived response (purple, *n* = 6 tumors), NR nonresponders (orange, *n* = 34 tumors). Line, single tumor. **f** Changes in non-synonymous single-nucleotide variant (nsSNV) numbers in paired pre- and on-treatment mouse samples from DR (*n* = 7 tumors) and NR (*n* = 6 tumors). Two-tailed Wilcoxon test was performed to determine statistical difference between matched pre- and on-treatment samples, two-tailed Mann–Whitney U test was performed to determine statistical difference between DR and NR groups in pre- or on-treatment samples. Dot, single tumor; line, paired samples; ns, not significant.

BRAF^V600E^ was expressed in the melanocytes of juvenile mice at ~8 weeks of age, and 4 weeks later, the mice were randomized to observation, or exposed to UVR for up to 26 weeks (Fig. 1a). Mice with established tumors (~100 mm^3^) were treated with anti-PD-1 or isotype control immunoglobulin (IgG) and monitored for tumor growth. Similar to metastatic melanoma patients (Supplementary Fig. 1a), mice bearing multiple primary tumors mounted a mixed response to anti-PD-1 treatment (Fig. 1b; Supplementary Fig. 1b, c). To remove the complexity caused by analyzing multiple tumors in individual mice, we assessed responses to anti-PD-1 in mice bearing single tumors, either by restricting UVR to ~1 cm^2^ of the skin or by not exposing the mice to UVR (Fig. 1c). Pre-treatment biopsies were taken, and the mice were randomized to treatment with anti-PD-1 or IgG control. The IgG-treated mice had a median on-treatment survival of 43 days and none survived beyond day 62, whereas anti-PD-1-treated mice had a median survival of 49 days and ~27% survived beyond 62 days (Fig. 1d). Thus as in patients^15,16^, single-agent anti-PD-1 produced a characteristic long-tail survival curve with significant benefit for the mice where tumor growth inhibition was observed (Fig. 1e; Supplementary Fig. 2a). Reflecting patient responses^17^, ~35% of the tumors from animals treated with anti-PD-1 responded to therapy: 13 tumors mounted durable responses (DR), 6 initially responded but then progressed (short-lived response; SR), and 34 tumors were nonresponsive (NR) and grew at similar rates to IgG-treated controls; these mice derived no survival benefit from the anti-PD-1 treatment (Fig. 1e; Supplementary Fig. 2a). Whole-exome sequencing (WES) of 28 tumors confirmed significantly increased single-nucleotide variant (SNV) burden in the UVR-induced tumors and no significant difference in indels associated with UVR exposure (Supplementary Fig. 2b, c). The observed responses to anti-PD-1 were independent of whether the animals received UVR or not (Supplementary Fig. 2d). As in melanoma patients^18^, for 13 mice where paired baseline and on-treatment WES was available, the sporadic changes in non-synonymous (ns)SNV load were independent of response (Fig. 1f). Also similar to melanoma patients^19^, the predicted neo-antigen load correlated with nsSNV burden, but alone did not determine prolonged response (Supplementary Fig. 2e, f). Together, our results demonstrate that UVR-induced SNVs in the context of a stable genome do not increase the odds of response to anti-PD-1 treatment.

### Influx of T cells is not required to maintain tumor response

**Fig. 2:**
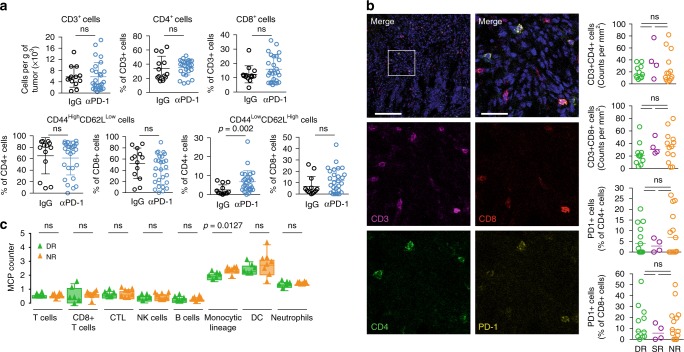
Maintenance of response is not associated with specific immune profile. **a** Flow cytometry analysis of T lymphocyte subpopulations in animals treated with IgG (black, *n* = 13 tumors) or anti-PD-1 (blue, *n* = 26 tumors). CD3^+^ cell count normalized to tumor weight (g), and relative abundance of CD4^+^ and CD8^+^ cells in the same tumors are shown (top). Abundance of effector/memory T cells (CD44^high^CD62L^low^) and naive T cells (CD44^low^CD62L^high^) among CD4^+^ and CD8^+^ cells (bottom). Two-tailed Mann–Whitney *U* test was performed to determine statistical difference between the treatment groups. Circle, single tumor; horizontal line, mean; error bar, standard deviation; ns, not significant. **b** Representative immunofluorescence images and quantification of mouse tumors co-stained with DAPI (blue), CD3 (magenta), CD4 (green), CD8 (red), and PD-1 (yellow). Scale bar, 100 μm (top left picture) or 25 μm (top right picture). DR, green, *n* = 11 tumors; SR, purple, *n* = 4 tumors; NR, orange, *n* = 13 tumors. Two-tailed Mann–Whitney *U* test was performed to determine statistical difference between the response groups. Circle, single tumor; line, mean; ns, not significant. **c** Abundance of eight immune cell types from RNA-seq analysis in DR (green, *n* = 6 tumors), and NR (orange, *n* = 8 tumors) using MCP-counter algorithm^22^. Two-tailed Mann–Whitney *U* test was performed to determine statistical difference between the response groups. Triangle, single tumor; hinges, 25th and 75th percentiles; middle line, median; whiskers, minimum to maximum value.

Defects in the IFNγ pathway and PD-L1 or MHC-II expression previously correlated with response to anti-PD-1 in patients^8,20^ were not observed in our NR mice (Supplementary Fig. 3a, b). However, we observed a trend toward increased PD-L1 expression in tumor cells (CD45^−^) and tumor-associated leukocytes (CD45^+^) consistent with activation of IFNγ signaling in anti-PD-1-treated animals (Supplementary Fig. 3c, d). Although we did observe a general increase in naïve T cells (CD44^low^CD62L^high^), anti-PD-1 did not induce significant changes in total T cells (CD3^+^) or in the relative abundance of CD4^+^, or memory/effector T cells (CD44^high^CD62L^low^; Fig. 2a; Supplementary Fig. 3e). Infiltration by CD8^+^ T cells was higher in animals that received anti-PD-1, however, no significant differences in T-cell infiltration was observed between DR and NR after prolonged therapy with PD-1 blockade (Fig. 2b). Taken together, our data suggest that in the late stages of therapy, sustained response to anti-PD-1 does not rely on the continuous influx or expansion of cytotoxic T cells.

We used RNAseq to characterize further immune cell infiltration into our mouse tumors^21,22^. Consistent with the experimentally controlled parameters of this closed system, the proportion of different cell types was stable across the samples, but no specific alterations correlated with durable response (Supplementary Fig. 4a; Supplementary Data 1). Lineage-specific gene signatures^22,23^ confirmed comparable levels of T cell, B cell, and macrophage markers in DR and NR tumors (Fig. 2c; Supplementary Fig. 4b; Supplementary Data 2), and flow cytometry for tumor-associated macrophages (F4/80^+^) and dendritic cells (F4/80^−^CD11c^+^) supported these findings (Supplementary Fig. 4c, d). Together our results demonstrate that no single immune cell type alone is responsible for the maintenance of response after prolonged treatment with anti-PD-1.

### Increase in CAFs characterizes durable responses to ICIs

**Fig. 3:**
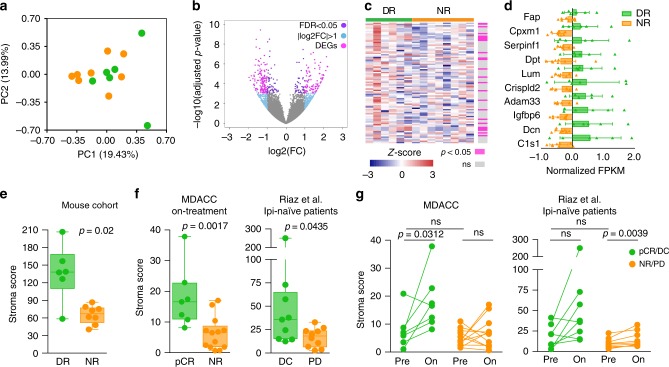
Stroma remodeling in melanomas with durable responses to ICIs. **a** Principal component analysis (PCA) plot of RNA-seq data from DR (green, *n* = 6 tumors) and NR (orange, *n* = 8 tumors). Dot, single tumor; PC, principal component. **b** Volcano plot of DEGs (differentially expressed genes, magenta) in DR and NR tumors with FDR (false discovery rate) < 0.05 and |log2FC| (absolute log2 fold change) >1. **c** Heatmap representing gene expression of previously reported lineage-specific markers^23^ of cancer-associated fibroblasts (CAFs) in DR (green, *n* = 6 tumors) and NR (orange, *n* = 8 tumors). Significantly differentially expressed genes (pink, *p* < 0.05) are highlighted using DESeq2 algorithm. Column, single tumor; row, gene; ns, not significant (gray). **d** Gene expression of the top ten CAF markers significantly upregulated in DR (green, *n* = 6 tumors) versus NR (orange, *n* = 8 tumors). FPKM values for each gene are normalized to the mean expression obtained from all tumors. Triangle, single tumor; bar, mean of the response group; error bar, standard deviation. **e** Stroma score generated from the 10-gene CAF signature in **d** for DR (green, *n* = 6 tumors) and NR (orange, *n* = 8 tumors). Two-tailed Mann–Whitney *U* test was performed to determine statistical difference between the response groups. Dot, single tumor; hinges, 25th and 75th percentiles; middle line, median; whiskers, minimum to maximum value. **f** Stroma score of melanoma samples from ICI-naive patients on-treatment with nivolumab monotherapy or in combination with ipilimumab from MDACC (left; pCR = 7 and NR = 13) and Riaz ipilimumab-naive^18^ (right; DC = 9 and PD = 10) cohorts. Two-tailed Mann–Whitney *U* test was performed to determine statistical difference between the response groups. Dot, single tumor; hinges, 25th and 75th percentiles; middle line, median; whiskers, minimum to maximum value. **g** Stroma score for paired pre- and on-treatment tumor samples from MDACC (left; pCR = 7 and NR = 12 patients) and Riaz ipilimumab-naive^18^ (right; DC = 8 and PD = 9 patients) cohorts. Two-tailed Wilcoxon test was performed to determine statistical difference between matched pre- and on-treatment samples, two-tailed Mann–Whitney *U* test was performed to determine statistical difference between response groups in pre- or on-treatment samples. pCR pathologic complete response, NR non-responder, DC disease control, PD progressive disease. Dot, single tumor; line, paired samples; ns, not significant.

Using principal component analysis (PCA) of RNA-seq data, we did not observe cluster separation (Fig. 3a), but we did identify 235 genes that were differentially expressed between DR and NR tumors (FDR < 0.05 and fold change > 2; Fig. 3b; Supplementary Data 3). Among these, 23 genes were previously reported to be expressed in cancer-associated fibroblasts (CAFs)^23^ (Fig. 3c; Supplementary Data 4) supporting an impact of anti-PD-1 therapy on the TME. By selecting the top ten upregulated genes from this set, we generated a stroma score that segregated DR from NR tumors (Fig. 3d, e). As RNA-seq data from patients with prolonged responses is unavailable, we evaluated this score in early on-treatment samples from melanoma patients receiving nivolumab monotherapy or combination nivolumab/ipilimumab from two independent cohorts. In a cohort from the MD Anderson Cancer Centre (MDACC)^24,25^, 23 ICI-naive patients received either combination nivolumab/ipilimumab (*n* = 11 patients) or single-agent nivolumab (*n* = 12 patients) in the neoadjuvant setting. RNA-seq data were available for 20 patients at 3 or 4 weeks of treatment (on-treatment), and from 17 patients’ surgical samples following treatment. Our stroma score significantly separated patients who achieved pathologic complete response (pCR) from non-responding (NR) patients in on-treatment samples. Consistent with this result, end of treatment surgery samples in pCR patients had a higher stromal score than NR (Fig. 3f; Supplementary Fig. 5a). Our stroma score also separated advanced-stage melanoma patients who achieved disease control (DC) from those with progressive disease (PD) in the cohort by Riaz et al.^18^ who received anti-PD-1 as first-line immunotherapy (i.e., ipi-naive; *n* = 19 patients; Fig. 3f). However, the score did not separate DC from PD patients in the entire cohort by Riaz et al.^18^, which included an additional 29 patients who progressed on ipilimumab before receiving anti-PD-1 (ipi-progressors; Supplementary Fig. 5b). Finally, our stroma score did not segregate responding (pCR/DC) from non-responding (NR/PD) patients in the pre-treatment MDACC (*n* = 19 patients) and Riaz et al.^18^ ipi-naive (*n* = 17 patients) cohorts, supporting that stroma remodeling (as measured by increased stroma score) occurs early during ICI therapy in responding patients (Fig. 3g). These findings suggest that treatment with ICIs exerts differential effects on the stromal compartment that is associated with tumor response and is limited to first-line therapy.

### Reduced cell division identifies response early on treatment

**Fig. 4:**
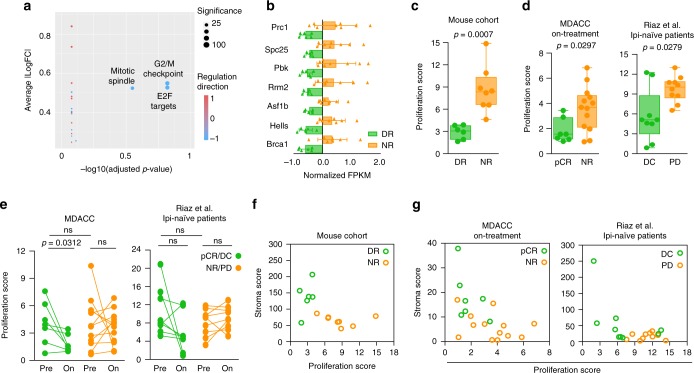
Tumor proliferation and stroma remodeling identify responding tumors early during treatment. **a** The top 20 differentially expressed hallmark signatures^36^ in RNAseq from DR (*n* = 6 tumors) and NR (*n* = 8 tumors) from mouse are reported in the plot. FC fold change. **b** Gene expression of the seven downregulated genes in DR vs. NR tumors included in the proliferation signature. FPKM values for each gene are normalized to the mean expression obtained from all tumors. Triangle, single tumor, bar, mean of the response group; error bar, standard deviation. **c** Proliferation score generated from the 7-gene signature in **b** for DR (green, *n* = 6 tumors) and NR (orange, *n* = 8 tumors). Two-tailed Mann–Whitney *U* test was performed to determine statistical difference between the response groups. Dot, single tumor; hinges, 25th and 75th percentiles; middle line, median; whiskers, minimum to maximum value. **d** Proliferation score of melanoma samples from ICI-naive patients on-treatment with nivolumab monotherapy or in combination with ipilimumab from MDACC cohort (left; pCR = 7 patients and NR = 13 patients) and Riaz ipilimumab-naive^18^ (right; DC = 9 patients and PD = 10 patients) cohorts. Two-tailed Mann–Whitney *U* test was performed to determine statistical difference between the response groups. Dot, single tumor; hinges, 25th and 75th percentiles; middle line, median; whiskers, minimum to maximum value. **e** Proliferation score for paired pre- and on-treatment samples from MDACC (left; pCR = 7 patients and NR = 12 patients) and Riaz ipilimumab-naive[18] (right; DC = 8 patients and PD = 9 patients) cohorts. Two-tailed Wilcoxon test was performed to determine statistical difference between matched pre- and on-treatment samples, two-tailed Mann–Whitney U test was performed for difference evaluation between response groups in pre- or on-treatment samples. Dot, single tumor; line, paired samples; ns, not significant. **f** Scatter plot of stroma vs. proliferation score for DR (green, *n* = 6 tumors) and NR (orange, *n* = 8 tumors) from mice treated with anti-PD-1. Circle, single tumor. **g** Scatter plot of stroma vs. proliferation score for DC and PD melanoma samples from MDACC (left; pCR = 7 patients and NR = 13 patients) and Riaz ipilimumab-naive^18^ (right; DC = 9 patients and PD = 10 patients) cohorts. Circle, single tumor. pCR, green, pathologic complete response; NR, orange, non-responder; DC, green, disease control; PD, orange, progressive disease.

To characterize further the overarching biological processes associated with the 235 DR and NR differentially expressed genes, we analyzed the distribution of curated collection of hallmark gene sets using gene set enrichment analysis. In line with previously published reports^26^, we observed an enrichment of genes associated with cell division downregulated in mice with DR on anti-PD-1 (Fig. 4a; Supplementary Fig. 6a; Supplementary Datas 5, 6). By merging the *G2M checkpoint* and the *E2F targets* programs, we developed a 7-gene proliferation score that separated our mice based on response (Fig. 4b, c). Thus, perhaps not surprisingly, our data suggest that responding tumors are characterized by reduced proliferation. However, the commonly used proliferation markers mitotic index and Ki67 staining lacked sufficient accuracy to segregate DR from NR tumors (Supplementary Fig. 6b, c). Similar to the stroma score, our proliferation score significantly differentiated responding (pCR/DC) from non-responding (NR/PD) patients in both the MDACC and Riaz et al.^18^ ipi-naive on-treatment cohorts (Fig. 4d). Consistent with this result, surgery samples from MDACC pCR patients had a lower proliferation score than samples from NR patients (Supplementary Fig. 6d). Like the stroma score, the proliferation score also did not separate responses in the entire 4-week on-treatment Riaz^18^ cohort (Supplementary Fig. 6e), or in pre-treatment biopsies from either the MDACC or Riaz^18^ cohorts (Fig. 4e). Interestingly, the simultaneous evaluation of the two processes by a combination score allowed complete segregation of the mice (Fig. 4f) and also distinguished responses in the ICI-naive patients after one cycle of therapy with sensitivity of 85.7% (6 of 7 patients) and 87.5% (7 of 8 patients), and specificity of 92.3% (12 of 13 patients) and 81.8% (9 of 11 patients) in the MDACC and Riaz et al.^18^ ipi-naive cohorts, respectively (Fig. 4g). In the MDACC time-of-surgery cohort, our combination score segregated pCR from NR patients with an accuracy of 75% sensitivity (Supplementary Fig. 6f). Our results suggest that a reduction in tumor proliferation early during treatment could identify which patients are more likely to achieve durable response with ICI.

## Discussion

Enormous variability within the human population, including genetics, environmental factors, and the diverse cellular composition of tumors impact how patients respond to ICIs. Consequently, massive sample sets are needed to identify patterns that reveal the biological responses while small differences go unnoticed. We therefore reasoned that because genetic and environmental variables are easily controlled in our BRAF^V600E^/UVR mouse melanoma model, we would be able to detect subtle changes in tumor and TME responses to immunotherapy, potentially providing better understanding of the human disease.

We previously reported that this model recapitulates the cardinal pathology and genomic features of human melanoma^12,14^, and show here that it also mimics human responses to ICI therapies with a tail of prolonged survival and examples of tumor progressing after initial response. Importantly, the distinct responses of single tumors that we observed within individual animals establish that response to therapy is not determined exclusively by the genetic background of the mouse, its UVR exposure, diet, or by the shaping of the immune system by the microbiome. Rather, our data show that prolonged responses occur through an intimate relationship between individual tumors, TME, and the immune system within an individual animal.

While our model recapitulates the increased SNVs burden resulting from UVR exposure seen in the human population^12,14^, chromosomal aberrations as well as insertions and deletions are rare. In this model, we did not observe significant association between UVR exposure and response to PD-1 blockade, but our data are concordant with previous reports showing a better correlation in response to ICI and high levels of microsatellite instability and indel load than to mutation burden^27,28^. In line with recently published results of a large meta-analysis in cancer patients^29^, PD-L1 expression status alone was not sufficient to segregate responding from non-responding animals. In addition, our model demonstrates that sustained response late during the course of therapy is not associated with specific immune profile, as opposed to earlier phases for which various immune signatures that distinguish responding from non-responding patients treated with ICIs have been described^6,30^.

As tumors develop, diverse cell types are recruited to the TME which can reduce anti-tumor immunity^31^. Analysis of RNA-seq data from our mice revealed significant difference in the expression of several cancer-associated fibroblast markers in responding compared with non-responding tumors. We speculate that under certain conditions, treatment with anti-PD-1 promotes the switch from an immunosuppressive TME characteristic of the tumor development and progression phase, to a more permissive state that favors T-cell trafficking into the tumor^11^. Consistent with previous studies^26^, reduced proliferation emerged as an important feature in responding tumors from our mice. Importantly, both the stroma and the proliferation scores identified in this closed system successfully anticipated early responses to ICI in all the currently available on-treatment human cohorts, validating our preclinical results in human cancer and supporting the use of representative mouse models to discover new biology in human disease. Finally, as suggested by previous studies^5,6^, we show that prior lines of therapy, and in particular selective pressure from treatment with ipilimumab alters both the tumor and TME characteristics thus masking the response to second-line anti-PD-1.

In summary, we used our mouse model to explore the biology of prolonged melanoma responses to ICI. Critically, the durable responses in our mice mirrored the early responses in two independent human cohorts, highlighting the power of relevant preclinical disease models for the identification of small or subtle changes in tumor biology. Our ability to predict within one cycle (3 or 4 weeks) of treatment which patients are likely to progress on single-agent anti-PD-1 could allow those patients to switch to more effective combination therapies, including those based on anti-CTLA-4. This could potentially provide those patients with the opportunity for more effective treatments, while saving the patients likely to mount a durable response to single-agent anti-PD-1 from the risk of the unnecessary toxicity associated with combination therapies. Apart from improving patient care, this could achieve considerable cost savings. Additional validation of the scores herein described in larger, ideally longitudinal patient cohorts would consolidate their use to guide treatment selection at an earlier stage than is currently possible with imaging. Further investigation of the tumor phenotypes associated with response to protracted ICI is needed to evaluate their potential for translation into reliable biomarkers for patient stratification and their possible application for establishing new rationally designed drug combinations. Clearly, this could improve the delivery of personalized immunotherapy to patients.

## Methods

### CT scans

Images from CT scan were obtained under the Manchester Cancer Research Centre (MCRC) Biobank ethics application #07/H1003/161 + 5 with full informed consent from the patient at The Christie NHS Foundation Trust. The work was approved by MCRC Biobank Access Committee application 13_RIMA_01. Response to treatment was assessed at 12–16 weeks by radiographic imaging using Response Evaluation Criteria In Solid Tumors, version 1.1 (RECIST v1.1).

### MDACC patients

NCT02519322 trial was approved by the MD Anderson Cancer Center Institutional Review Board. The trial was conducted in accordance with the ethical principles of the Declaration of Helsinki and with adherence to the Good Clinical Practice guidelines, as defined by the International Conference on Harmonization. The protocol was conducted with compliance with all relevant ethical regulations and written informed consent was obtained from all participants.

### Animal work

All procedures involving animals were performed under the Home Office approved project license PE3DF1A5B, in accordance with ARRIVE guidelines and UK Home Office regulations under the Animals (Scientific Procedures) Act 1986. The study received ethical approval by the Cancer Research UK Manchester Institute’s Animal Welfare and Ethics Review Body (AWERB). Tyr::CreER^T2^,Braf^V600E^ (C57BL/6 background) were bred in a specific pathogen-free facility at The University of Manchester (UK). All mice were maintained in pathogen-free, ventilated cages in the Biological Resources Unit at Cancer Research UK Manchester Institute, and allowed free access to irradiated food and autoclaved water in a 12 h light/dark cycle, with room temperature at 21 ± 2 °C. All cages contained wood shavings, bedding and a cardboard tube for environmental enrichment. Female mice, 8–10 weeks of age were enrolled into the experiments. Mice were monitored daily and euthanized by cervical dislocation prior to any signs of distress or when their cumulative tumor burden reached a maximum of 1500 mm^3^ determined by caliper measurements. All the procedures were conducted in the light phase. BRAF^V600E^ was induced by tamoxifen (Sigma-Aldrich; #T5648) freshly prepared in 100% ethanol applied topically to the shaven back of juvenile mice. Four weeks later, mice were randomized to either weekly exposure to UVR (full back exposure Fig. 1b, Supplementary Fig. S1B, S1C; or ~1 cm^2^ otherwise) or monitored until tumor formation. Tumor volume was determined by caliper measurements of tumor length (*L*), width (*W*), and depth (*D*), and calculated as volume = *L* × *W* × *D* × π/6. Pre-treatment tumor biopsies were obtained when tumors reached ~100 mm^3^. Prior to surgery, mice were given a subcutaneous injection of 10 mg kg^−1^ Carprophen (Rimadyl) as analgesic. Tumor biopsies were taken under inhalation anesthesia (isoflurane 2–3%) in class II cabinets. One week after biopsy, mice were randomized into two treatment groups receiving either anti-PD-1 (BioXcell; #BE0146; clone RMP1-14; 10 mg kg^−1^ twice weekly by i.p. injection) or rat IgG2a (BioXcell; #BE0089; clone 2A3; 10 mg kg^−1^ twice weekly by i.p. injection). Survival was analyzed in mice bearing single tumors starting on the day the treatment commenced until tumor volume reached 1500 mm^3^. Animals were censored when sacrifice during treatment was independent of tumor growth. The experiment was repeated, and data were pooled for all the analyses.

### Whole-exome sequencing

Snap-frozen tumor tissue was manually dissected by sectioning (25 μm thick), and DNA was extracted from sections with an estimated tumor cell percentage of at least 80% using AllPrep DNA/RNA kit (Qiagen) according to the manufacturer’s instructions. Germline DNA was isolated from mouse kidney. DNA quality was assessed using a Qubit^®^ 2.0 Fluorometer (Life Technologies). Exome capture was performed using the Agilent SureSelect Mouse All Exon V1 with 200 ng of genomic DNA, according to the manufacturer’s instructions. WES was performed on the Illumina HiSeq 2500 with read length of 2 × 100 bps. After removing adapters using Cutadapt (v1.14) and trimming poor quality base calls using Trimmomatic (v0.36), the reads were aligned to the GRCm38 (release mm10) mouse genome using BWA aligner (v0.7.7). The PCR duplicate reads were filtered using Picard (v1.96), and the base quality score recalibration and local INDEL realignments were performed using GATK tools (v3.1). Using tumor-normal pairs, SNVs and indels were identified using VarScan 2^32^ (v2.3.6). Variant effect predictor (Ensembl version 73/84) was used to annotate the mutations. Known variants present in dbSNP were excluded.

### Neo-antigens prediction

A comprehensive list of peptides (9–11 amino acids in length) was generated using missense mutations such that the peptide list contained mutated amino acid in each possible position. The binding affinities of the mutant and corresponding wild-type peptide to the H2-Kb mouse alleles were predicted using netMHCpan 4.0 web server^33^. Peptides with predicted binding strength <500 nM were considered as candidate neo-antigens.

### Flow cytometry

Tumors were minced and digested by using the mouse Tumor Dissociation Kit (Miltenyi Biotech), washed with FACS buffer (PBS containing 2% FBS, 2 mM EDTA and 0.02% sodium azide), and filtered through a 70 -µm filter (BD Biosciences). The obtained single-cell suspension was stained with LIVE/DEAD Fixable Blue Dead Cell Stain kit (Thermo Fisher Scientific), blocked with anti-CD16/32 (BD Biosciences; #553142; clone 2.4G2; 1:1000), stained with fluorochrome-labeled antibodies, and analyzed using a LSR Fortessa (BD Biosciences) and FlowJo software (Tree Star Inc).

The following antibodies were purchased from BD Biosciences: CD3-BUV737 (#564380; clone 17A2; 1:400), CD8a-BB515 (#564422; clone 53-6.7; 1:400), CD44-APC-Cy7 (#560568; clone IM7; 1:1000), CD274-BV711 (#563369; clone MIH5; 1:200), CD11b-FITC (#557672; clone M1/70; 1:800), CD45-BV605 (#563053; clone 30F11; 1:400). The following antibodies were purchased from Biolegend: CD4-BV510 (#100449; clone GK1.5; 1:400), CD62L-BV785 (#104440; clone MEL-14; 1:200), CD161-PE (#108707; clone PK136; 1:400), CD11c-PE (#117308; clone N418; 1:200), F4/80-AF647 (#123122; clone BM8; 1:2000).

### Immunofluorescence

Mouse tissue was embedded in optimal cutting temperature (O.C.T.) compound (Thermo Fisher Scientific), snap-frozen and sectioned (7 μm) on a cryostar NX70 cryostat (ThermoShandon). Following fixation (acetone/methanol), sections were incubated in 5% normal goat serum followed by primary antibody incubation (1 h at room temperature). The following anti-mouse primary antibodies from Thermo Fisher Scientific were used: CD3-eFluor660 (#50–0032; clone 17A2, 1:200), CD4-eFluor570 (#41–0042; clone RM4-5; 1:50), and CD8a-eFluor615 (#42–0081; clone 53-6.7; 1:50). Anti-mouse PD-1 (clone RMP1-14; 7.2 mg ml^−1^, 1:500) was produced in house by Louis Boon. This was detected by AlexaFluor488-conjugated anti-rat IgG secondary antibody (Stratech Scientific; #712-545-153; 1:400). Sections were then counterstained with DAPI and coverslipped with prolong gold (Thermo Fisher Scientific).

### Imaging analysis

Tumor sections stained for multiplexed lymphocyte markers were imaged with a Leica TCS SP8 gSTED-FLIM-FCS microscope. A minimum of eight fields of view was captured per section, depending on section size. Positive lymphocytes for the indicated markers and total cell number (DAPI) per field were automatically counted using Imaris v9 (Bitplane). Data are presented as total lymphocytes count per mm^2^ of tumor section.

### RNA sequencing of mouse tumors

RNA was extracted from fresh frozen tumor samples using AllPrep DNA/RNA kit (Qiagen) according to the manufacturer’s instructions. Indexed PolyA libraries were prepared using 200 ng of the total RNA and 14 cycles of amplification with the Agilent SureSelect Strand-Specific RNA Library Prep Kit for Illumina Sequencing (Agilent, G9691A). Libraries were quantified by qPCR using a Kapa Library Quantification Kit for Illumina sequencing platforms (Kapa Biosystems Inc., 230 KK4835). The analysis was performed on Illumina HiSeq 2500 with pair-end reads (2 × 100). After removing adapters using Cutadapt (v1.14) and trimming poor quality base calls using Trimmomatic (v0.36), the reads were aligned to GRCm38 (release 86) using STAR aligner (v2.5.1)^34^. Gene counts were subsequently estimated using StringTie (v1.3.1)^35^. After removing transcripts without minimum 1 read in at least three samples, the differential expression analysis between mice that responded and nonresponders was performed using DESeq2 (v1.14.1)^34^. The resultant *p*-values were corrected for multiple comparisons using the Benjamini–Hochberg approach. The principal component plots and heatmaps were generated using pheatmap package (v1.0.8) on log-transformed DESeq2-normalized counts. We used EGSEA package (v1.2.0)^36,37^ with Limma-based expression analysis to calculate single-sample gene set enrichment (ssGSEA) on Hallmark gene sets^38^.

### RNA sequencing of MDACC human samples

The total RNA was extracted from snap-frozen macrodissected tumors using the AllPrep DNA/RNA/miRNA Universal Kit (Qiagen). RNA quality was assessed on an Agilent 2100 Bioanalyzer using the Agilent RNA 6000 Nano Chip. The total RNA (40–80 ng) was used for library preparation with the Illumina TruSeq RNA Access Library Prep kit, according to the manufacturer’s protocol. Barcoded libraries were pooled to produce final 10–12 plex pools prior to sequencing on an Illumina NextSeq sequencer using one high-output run per pool of 76 bp paired-end reads, generating 8 fastq files (4 lanes, paired reads) per sample. Quality control of the FASTQ files was first performed through FastQC (v0.11.5)^39^. Reads with 15 contiguous low-quality bases (phred score < 20) were removed. STAR 2-pass alignment (v2.5.3)^40^ with default parameters was then performed to generate the BAM files. RNA-SeQC (v1.1.8)^41^ was run on the BAM files to evaluate read counts, coverage, and correlation. A matrix of Spearman correlation coefficients was generated by RNA-SeQC, and one library pool that had poor correlation with other pools from the same sample was removed before sample-level merging of BAM files. Aligned RNA-seq BAM files were processed through HTSeq-count (v0.9.1)^42^ tool, and the raw counts were normalized into fragments per kilobase of transcript per million mapped reads (FPKM) using the NCI Genomic Data Commons guidelines (GDC)^43^. Surgical samples were analyzed by dermatopathologists for pathologic complete response (pCR), defined as absence of any viable malignant cells on hematoxylin and eosin stained slides, all the other patients were classified as nonresponders (NR)^24,25^.

### Analysis of RNA sequencing data from Riaz et al

The raw counts from RNA sequencing analysis from Riaz et al.^18^ were downloaded from GEO (accession number GSE91061). Patient 3 was excluded from the analysis, in agreement with the authors’ annotations. Pheatmap was used to generate various heatmaps from the log-transformed normalized raw counts from DESeq2. Response to treatment was defined by RECIST v1.1 as complete response, partial response and stable disease, all of which were included in the disease control (DC) group in this study, and progressive disease (PD).

### Immune cell infiltrate analysis

The ImmuCC^21^ algorithm was used for the immune deconvolution on the FPKM values of mouse tumors. Absolute quantification of immune cell types was computed using MCP-counter (v1.1.0)^22^. The mouse orthologs of specific markers for T cells, B cells, macrophages, and CAFs^23^ were used for analysis of the immune and stroma infiltrate.

### Proliferation and Stroma score

Unique genes from merged G2M checkpoint and E2F targets pathways were selected based on absolute log2 fold change ≥1 (from DESeq2 analysis) between responders and nonresponders. The product of this analysis constituted the proliferation score. Within the CAF signature^23^, the top ten genes with statistically significant upregulation in responding tumors (unadjusted *p*-value) constituted the stroma signature. The final proliferation and stroma scores were calculated by taking geometric means of respective gene sets.

### Histology and immunohistochemistry

Sections were deparaffinized with xylene and hydrated with a series of graded alcohol washes. Sections were microwaved in citrate buffer (pH 6) for antigen retrieval and rinsed in PBS washes. Sections were blocked in 1% BSA in PBS, incubated with 1:100 dilution of rabbit anti-Ki67 (Bethyl Laboratories; #IHC-00375) and secondary anti-rabbit (Dako; #K4003), and counterstained with hematoxylin. Mitotic index was calculated as the number of mitoses identified by morphological characteristics in an area of 1 mm^2^ within the region containing the highest mitotic density of the tumor, in a conventional H&E-stained slide. The result on Ki67 is presented as the percentage of tumor cells with positive nuclear staining for the marker. Both parameters are presented as average of the scoring from two independent pathologists.

### Statistical analyses

The statistical differences between two groups were assessed using two-tailed Mann–Whitney *U* test on GraphPad Prism version 7. Two-tailed Wilcoxon test was performed to determine statistical difference between matched pre and on-treatment samples with GraphPad Prism. Kaplan–Meier plots with the log-rank test were used to analyze survival data while *p*-values < 0.05 were considered significant.

### Reporting summary

Further information on research design is available in the Nature Research Reporting Summary linked to this article.

## Supplementary information

Peer Review File

Supplementary Information

Description of Additional Supplementary Files

Supplementary Data 1

Supplementary Data 2

Supplementary Data 3

Supplementary Data 4

Supplementary Data 5

Supplementary Data 6

Reporting Summary

## Data Availability

RNA sequencing and whole-exome sequencing data from mouse samples that support the findings of this study (Figs. 1–4; Supplementary Figs. 2–6) have been deposited in the European Nucleotide Archive (ENA) with accession code PRJEB35895. Sequencing data from MDACC human cohort that support the findings of this study (Figs. 3, 4; Supplementary Figs. 5, 6) have been deposited in the European Genome-phenome Archive (EGA) with accession code EGAS00001003178. The genetically engineered BRAF^V600E^ mouse strain is available from the corresponding author upon request. All other relevant data are available in the article, supplementary information, or available from the corresponding author on reasonable request.
