# Supplementary material for: Stroma remodeling and reduced cell division define durable response to PD-1 blockade in melanoma
Source: Nat Commun. 2020 Feb 12;11:853. doi: 10.1038/s41467-020-14632-2 (PMC7015935; doi:10.1038/s41467-020-14632-2)
Supplement: Supplementary file 2 — Supplementary Information [file 41467_2020_14632_MOESM2_ESM.pdf]

Stroma remodeling and reduced cell division define durable response to PD-1 blockade in melanoma.

Galvani, Mundra et al.

Supplementary Figures

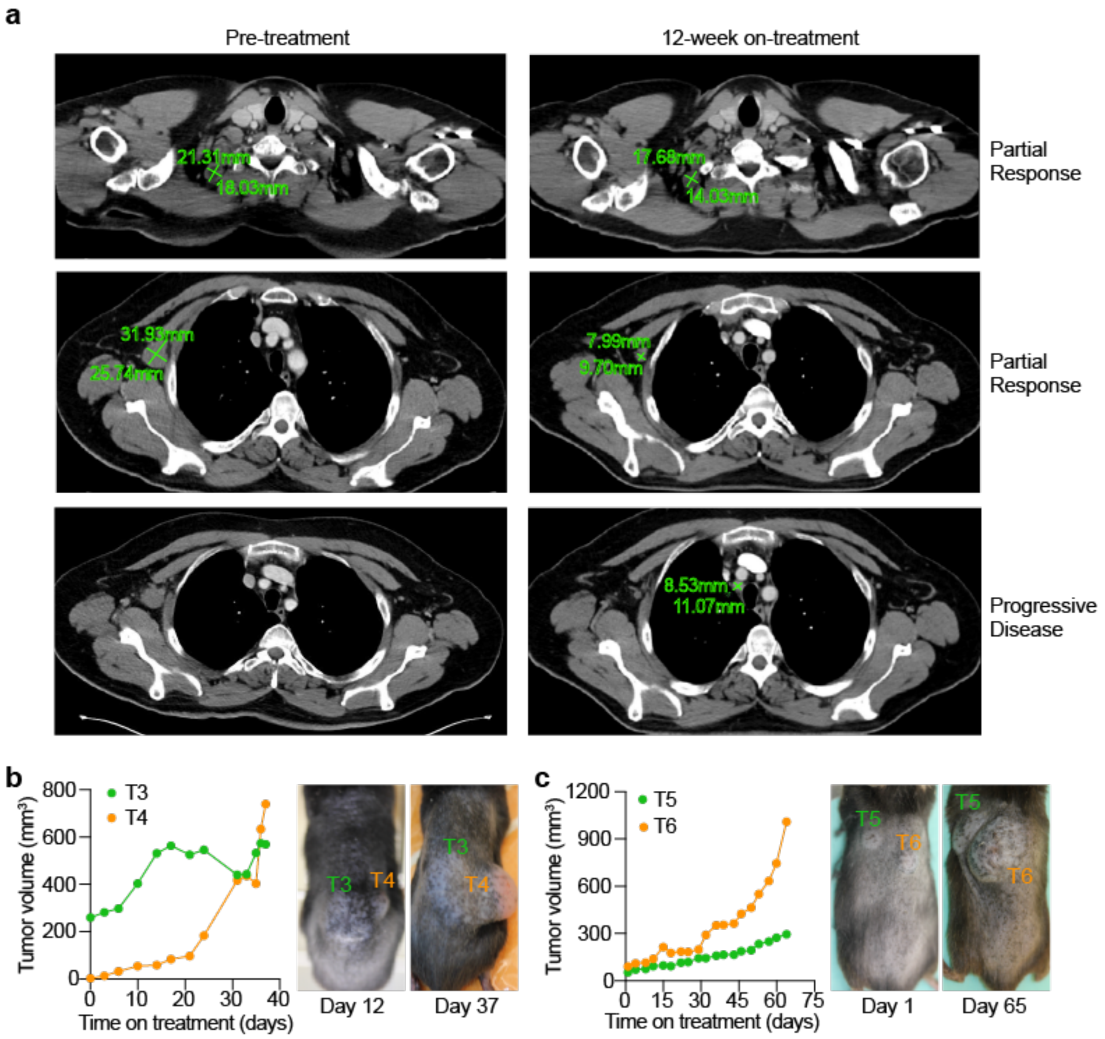

Supplementary Figure 1. Mixed response to anti-PD-1 in BRAF^{V600E} mice mirrors clinical response in melanoma patients. a Computed tomography (CT) scans from a representative case of mixed patient response to treatment with first-line anti-PD-1. Images of the differentially responding metastases (partial responses in the right supraclavicular and right axillary nodes at the top, and progressive disease in a right paratracheal node at the bottom) were taken pre-treatment and at week 12 on-treatment. **b,c** Growth curves and photographs at different treatment times of representative mice exposed **b** or not **c** to ultraviolet radiation (UVR) and bearing multiple tumors with differential response to PD-1 blockade.

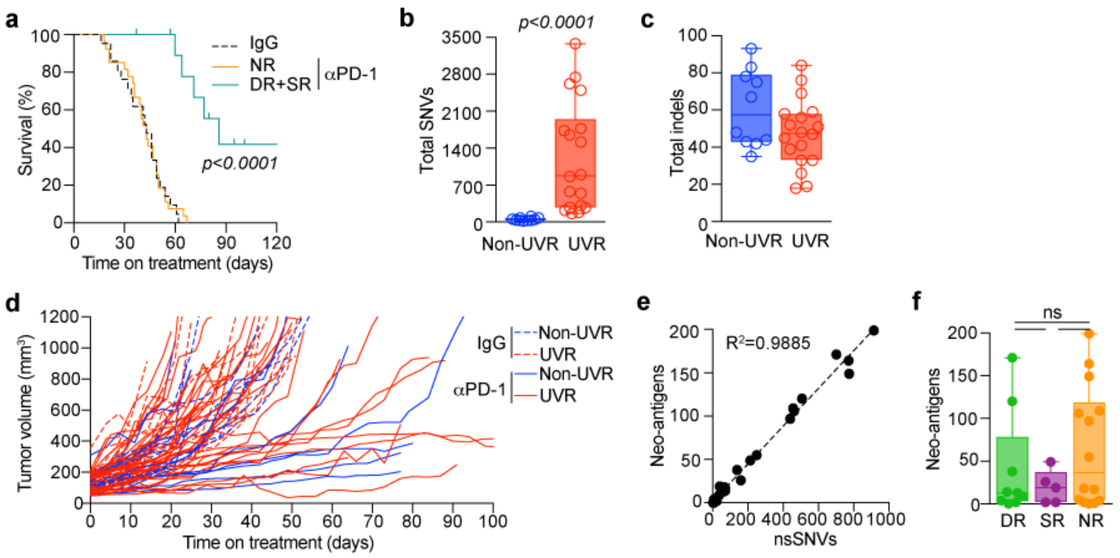

Supplementary Figure 2. PD-1 blockade results in survival benefit in mice. a Kaplan-Meier curves showing survival of animals treated with anti-PD-1, separated into non-responders (NR, *n*=27 animals; orange line) or durable + short-lived response (DR+SR, *n*=11 animals; green line), compared to IgG treated mice (*n*=21 animals; black dotted line); censored mice in the anti-PD-1 responding group were sacrificed for reasons other than tumor growth. Two-sided log-rank test was performed to determine statistical difference between the survival curves. **b,c** Number of total single nucleotide variants (SNVs) **b** and indels **c** in non-UVR (*n*=10 tumors) or UVR (*n*=18 tumors) samples. Two-tailed Mann Whitney U test was performed to determine statistical difference between the groups. **d** Tumor growth curves from Fig. 1e colored according to exposure (red) or not (blue) to ultraviolet radiation (UVR). Line, single tumor. **e** Pearson correlation between number of non-synonymous (ns) SNVs and predicted neo-antigens in *n*=28 mouse tumors. Dot, single tumor; line, best fitted curve. **f** Number of predicted neoantigens in samples from DR (green, *n*=9 tumors), SR (purple, *n*=5 tumors) and NR (orange, *n*=14 tumors). Two-tailed Mann Whitney U test was performed to determine statistical difference between the response groups. Dot, single tumor; hinges, 25th and 75th percentiles; middle line, median; whiskers, minimum to maximum value; ns, not significant.

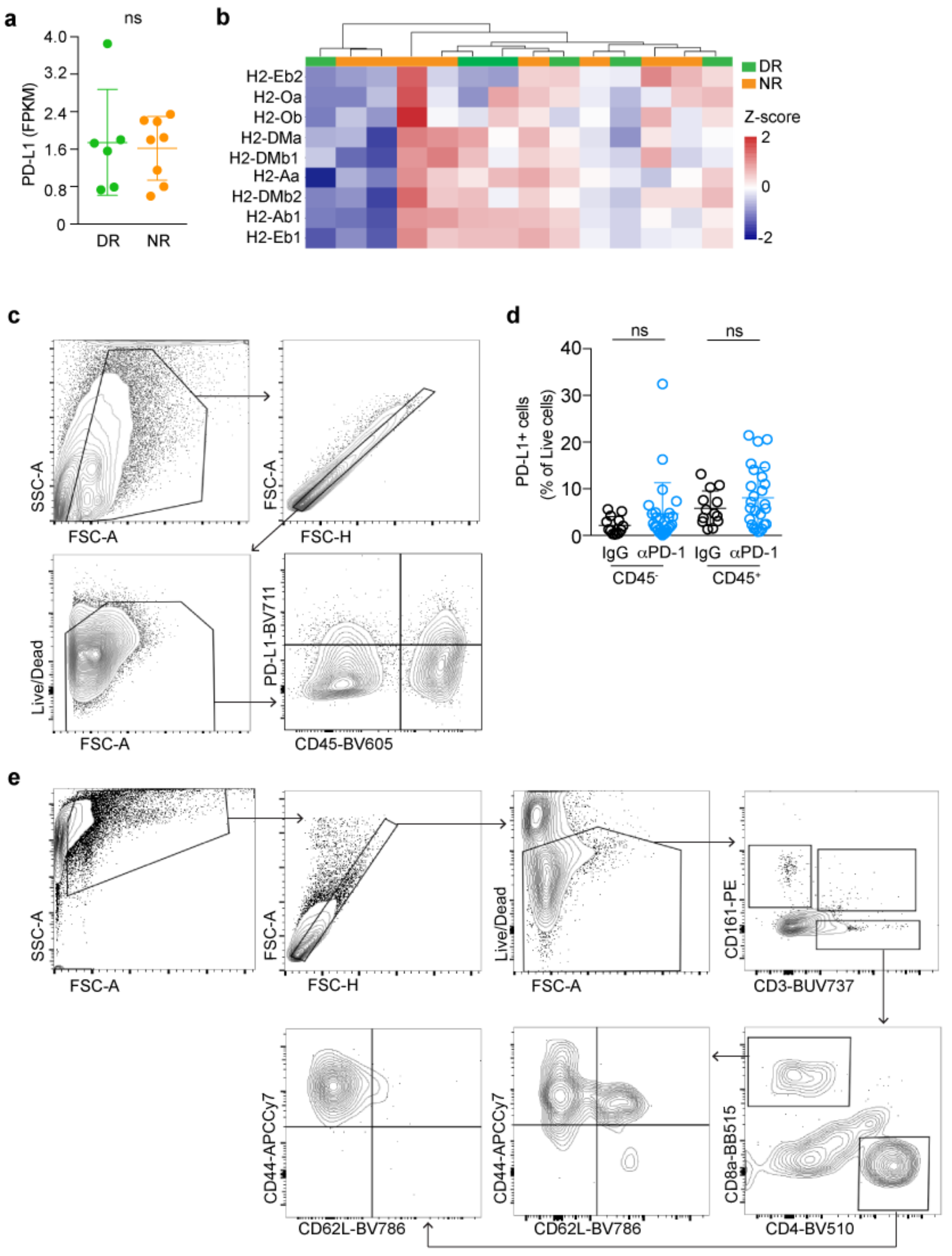

Supplementary Figure 3. PD-L1 and MHC-II expression do not distinguish responding from non-responding melanoma in mice. a PD-L1 gene expression in DR (green, *n*=6 tumors) and NR (orange, *n*=8 tumors) obtained from RNAseq. Two-tailed Mann Whitney U test was performed to determine statistical difference between the response groups. Dot, single tumor; line, mean; error bar, standard deviation; ns, not significant. **b** Heatmaps representing gene expression of several MHC-II molecules in DR (green, *n*=6 tumors) and NR (orange, *n*=8 tumors) from RNAseq. Row, gene; column, sample. **c** Gating strategy to determine the percentage of tumor cells (CD45$^-$) and leucocytes (CD45$^+$) expressing PD-L1. **d** Percentage of PD-L1 expressing tumor and immune cells in IgG (*n*=13 tumors) and anti-PD-1 (*n*=26 tumors) treated mice. Two-tailed Mann Whitney U test was performed to determine statistical difference between the treatment groups. Circle, single tumor; line, mean; error bar, standard deviation; ns, not significant. **e** Gating strategy to determine the percentage of T cells subgroups infiltrating the tumors in IgG (*n*=13 tumors) and anti-PD-1 (*n*=26 tumors) treated mice (Fig. 2a).

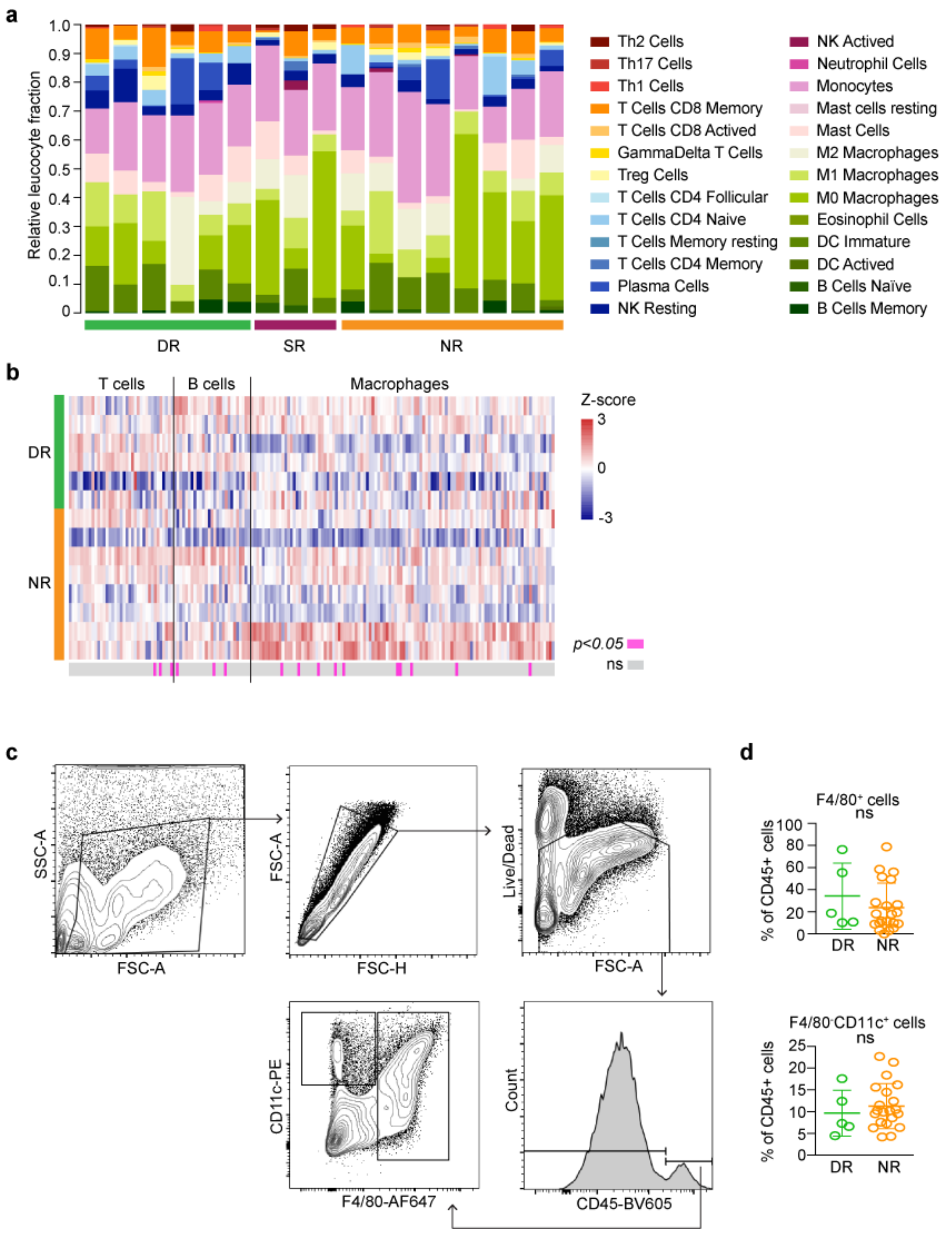

Supplementary Figure 4. Tumor immune infiltrate does not correlate with response in mice. a Relative abundance of 26 immune cell types from gene expression analysis by ImmuCC pipeline[21] in DR (green, *n*=6 tumors), SR (purple, *n*=3 tumors), and NR (orange, *n*=8 tumors). Column, single tumor. **b** Heatmap representing the gene expression of lineage specific T cells, B cells and macrophage markers[23] in DR (green, *n*=6 tumors) and NR (orange, *n*=8 tumors). Significantly differentially expressed genes (pink, $p<0.05$) are highlighted using DESeq2 algorithm. Column, gene; row, single tumor. **c** Gating strategy to determine the percentage of tumor associated macrophages (F4/80$^+$) and dendritic cells (F4/80$^-$CD11c$^+$) in mouse tumors. **d** Relative abundance of F4/80$^+$ (top) and F4/80$^-$CD11c$^+$ (bottom) cells in DR (green, *n*=5 tumors) or NR (orange, *n*=21 tumors). Two-tailed Mann Whitney U test was performed to determine statistical difference between the response groups. Dot, single tumor; horizontal line, mean; error bar, standard deviation. ns, not significant.

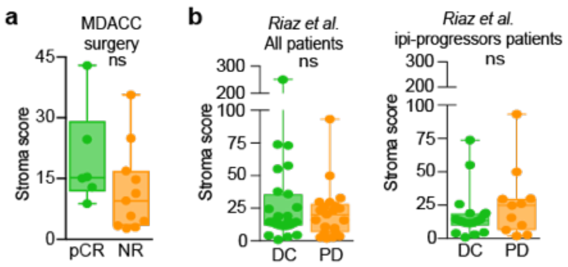

Supplementary Figure 5. Changes in the stroma compartment correlate with response in first line ICI treated patients. a Stroma score generated from the 10-gene CAF signature for melanoma samples from patients who received combination nivolumab+ipilimumab or nivolumab monotherapy in the neoadjuvant setting. RNAseq from samples obtained at the time of surgery in the MDACC cohort. Patients with pathologic complete response (pCR): *n*=6 patients, green; non-responding patients (NR): *n*=11 patients, orange. Two-tailed Mann Whitney U test was performed to determine statistical difference between the response groups. **b** Stroma score generated from the 10-gene CAF signature in the Riaz cohort[18] of melanoma patients with disease control (DC) or progressive disease (PD) on-treatment with anti-PD-1 (entire cohort on the left; DC, *n*=27 patients; PD, *n*=21 patients) or in patients who received prior treatment with ipilimumab (right; DC, *n*=18 patients; PD, *n*=11 patients). Two-tailed Mann Whitney U test was performed to determine statistical difference between the response groups. Dot, single tumor; hinges, 25th and 75th percentiles; middle line, median; whiskers, minimum to maximum value; ns, not significant.

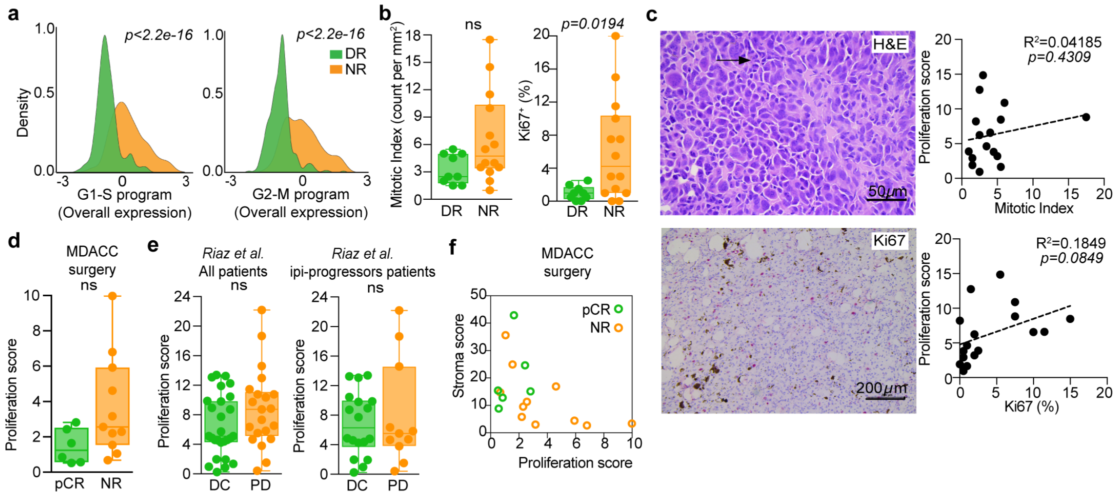

Supplementary Figure 6. Common markers of proliferation do not segregate responders and non-responders to anti-PD-1 treatment. a Overall gene expression profile of DR (green, *n*=6 tumors) and NR (orange, *n*=8 tumors) of the G1-S and G2-M programs[23]. The p-values are determined using Welch's two sample t-test. **b** Mitotic index (left) and percentage of Ki67 positive cells (right) in DR (green, *n*=9 tumors) and NR (orange, *n*=14 tumors) from mice treated with anti-PD-1 assessed in hematoxylin and eosin (H&E) or immunohistochemistry staining, respectively. Plotted values represent the mean of the scores obtained from two independent pathologists blinded to the tumor response to therapy. Two-tailed Mann Whitney U test was performed to determine statistical difference between the response groups. Dot, single tumor; hinges, 25th and 75th percentiles; middle line, median; whiskers, minimum to maximum value; ns, not significant. **c** Representative images of H&E and immunohistochemistry staining for the evaluation of mitotic index and Ki67 (in red), respectively (left). Pearson correlation (right) between mitotic index (top) or Ki67 positivity (bottom) and proliferation score for *n*=17 tumors. Dot, single tumor; line, best fitted curve. **d** Proliferation score generated from the 7-gene signature for melanoma samples from patients who received the combination nivolumab+ipilimumab or nivolumab monotherapy in neoadjuvant setting. RNAseq from samples obtained at the time of surgery in the MDACC cohort. Patients with pathologic complete response (pCR): *n*=6, patients, green; non-responding patients (NR): *n*=11 patients, orange. Two-tailed Mann Whitney U test was performed to determine statistical difference between the response groups. Dot, single tumor; hinges, 25th and 75th percentiles; middle line, median; whiskers, minimum to maximum value; ns, not significant. **e** Proliferation score generated from the 7-gene signature for melanoma samples from patients on-treatment with anti-PD-1 in the Riaz entire cohort[18] (left; DC, *n*=27 patients; PD, *n*=21 patients) or in patients who received prior treatment with ipilimumab (right; DC, *n*=18 patients; PD, *n*=11 patients). Two-tailed Mann Whitney U test was performed to determine statistical difference between the response groups. Dot, single tumor; hinges, 25th and 75th percentiles; middle line, median; whiskers, minimum to maximum value; ns, not significant. **f** Scatter plot of stroma *vs.* proliferation score in pCR (green, *n*=6 samples) and NR (orange, *n*=11 samples) melanoma patients from the MDACC cohort at the time of surgery. Circle, single tumor.
