# Supplementary material for: Stroma remodeling and reduced cell division define durable response to PD-1 blockade in melanoma
Source: Nat Commun. 2020 Feb 12;11:853. doi: 10.1038/s41467-020-14632-2 (PMC7015935; doi:10.1038/s41467-020-14632-2)
Supplement: Supplementary file 3 — Description of Additional Supplementary Files [file 41467_2020_14632_MOESM3_ESM.pdf]

Description of Additional Supplementary Files

File Name: Supplementary Data 1

Description: Results of immune cell infiltrate analysis by ImmuCC algorithm (Ref 22) on RNAseq data from mouse tumors relative to Supplementary Figure 4a.

File Name: Supplementary Data 2

Description: List of differentially expressed genes (DEGs) of lineage specific markers of T cells, B cells and macrophages (Ref. 23) between DR and NR relative to Supplementary Figure 4b.

File Name: Supplementary Data 3

Description: List of the differentially expressed genes (DEGs) between DR and NR mouse tumors relative to Figure 3b.

File Name: Supplementary Data 4

Description: List of differentially expressed genes (DEGs) of lineage specific markers of cancer associated fibroblasts (CAFs) (Ref. 23) between DR and NR relative to Figure 3c.

File Name: Supplementary Data 5

Description: Gene expression of the genes included in the G1-S program as identified by Tirosh and colleagues (Ref. 23) relative to Supplementary Figure 6a.

File Name: Supplementary Data 6

Description: Gene expression of the genes included in the G2-M program as identified by Tirosh and colleagues (Ref. 23) relative to Supplementary Figure 6a.
