# Supplementary material for: Stroma remodeling and reduced cell division define durable response to PD-1 blockade in melanoma
Source: Nat Commun. 2020 Feb 12;11:853. doi: 10.1038/s41467-020-14632-2 (PMC7015935; doi:10.1038/s41467-020-14632-2)
Supplement: Supplementary file 10 — Reporting Summary [file 41467_2020_14632_MOESM10_ESM.pdf]

nature research

Corresponding author(s): Richard Marais

Last updated by author(s): Jan 7, 2020

Reporting Summary

Nature Research wishes to improve the reproducibility of the work that we publish. This form provides structure for consistency and transparency in reporting. For further information on Nature Research policies, see Authors & Referees and the Editorial Policy Checklist.

Statistics

For all statistical analyses, confirm that the following items are present in the figure legend, table legend, main text, or Methods section.

n/a	Confirmed	
☐	☒	The exact sample size (*n*) for each experimental group/condition, given as a discrete number and unit of measurement
☐	☒	A statement on whether measurements were taken from distinct samples or whether the same sample was measured repeatedly
☐	☒	The statistical test(s) used AND whether they are one- or two-sided<br>*Only common tests should be described solely by name; describe more complex techniques in the Methods section.*
☒	☐	A description of all covariates tested
☐	☒	A description of any assumptions or corrections, such as tests of normality and adjustment for multiple comparisons
☐	☒	A full description of the statistical parameters including central tendency (e.g. means) or other basic estimates (e.g. regression coefficient) AND variation (e.g. standard deviation) or associated estimates of uncertainty (e.g. confidence intervals)
☐	☒	For null hypothesis testing, the test statistic (e.g. *F*, *t*, *r*) with confidence intervals, effect sizes, degrees of freedom and *P* value noted<br>*Give P values as exact values whenever suitable.*
☒	☐	For Bayesian analysis, information on the choice of priors and Markov chain Monte Carlo settings
☒	☐	For hierarchical and complex designs, identification of the appropriate level for tests and full reporting of outcomes
☐	☒	Estimates of effect sizes (e.g. Cohen's *d*, Pearson's *r*), indicating how they were calculated

Our web collection on statistics for biologists contains articles on many of the points above.

Software and code

Policy information about availability of computer code

Data collection	Flow cytometry data was collected by BD FACSDiva v7 (BD Biosciences). Remaining data was collected in Microsoft Excel Spreadsheets.
Data analysis	For WES: After removing adapters using Cutadapt (v1.14) and trimming poor quality base calls using Trimmomatic (v0.36), the reads were aligned to the GRCm38 (release mm10) mouse genome using BWA aligner (v0.7.7). The PCR duplicate reads were filtered using Picard (v1.96), and the base quality score recalibration and local INDEL realigments were performed using GATK tools (v3.1). Using tumour-normal pairs, SNVs and indels were identified using VarScan (v2.3.6). Variant Effect Predictor (Ensembl version 73/84) was used to annotate the mutations. For neo-antigens prediction: a comprehensive list of peptides (9-11 amino acids in length) was generated using missense mutations such that the peptide list contained mutated amino acid in each possible position. The binding affinities of the mutant and corresponding wild-type peptide to the H2-Kb mouse alleles were predicted using netMHCpan 4.0 web server. Peptides with predicted binding strength <500 nM were considered as candidate neo-antigens. For mouse RNAseq: after removing adapters using Cutadapt (v1.14) and trimming poor quality base calls using Trimmomatic (v0.36), reads were aligned to GRCm38 (release 86) using STAR aligner (v2.5.1). Gene counts were subsequently estimated using StringTie (v1.3.1). After removing transcripts without minimum 1 read in at least three samples, the differential expression analysis between mice that responded and non-responders was performed using DESeq2 (v1.14.1). The resultant p-values were corrected for multiple comparisons using the Benjamini-Hochberg approach. The principal component plots and heatmaps were generated using pheatmap package (v1.0.8) on log transformed DESeq2 normalized counts. We used EGSEA package (v1.2.0) with Limma based expression analysis to calculate single-sample gene set enrichment (ssGSEA) on Hallmark gene sets. For human RNAseq: quality control of the FASTQ files was first performed through FastQC (v0.11.5). Reads with 15 contiguous low-quality bases (phred score<20) were removed. STAR 2-pass alignment (v2.5.3) with default parameters was then performed to generate the BAM files. RNA-SeQC (v1.1.8) was run on the BAM files to evaluate read counts, coverage, and correlation. A matrix of Spearman correlation coefficients was generated by RNA-SeQC and one library pool that had poor correlation with other pools from the same sample was removed before sample-level merging of BAM files. Aligned RNA-seq BAM files were processed through HTSeq-count (v0.9.1) tool and the raw counts were normalized into fragments per kilobase of transcript per million mapped reads (FPKM) using the NCI Genomic Data Commons guidelines (GDC). Immune cell infiltrate: The previously published ImmuCC algorithm was used for the immune deconvolution on the FPKM values of mouse tumour. Absolute quantification of immune cell types was computed using MCP-counter (v1.1.0). The mouse orthologs of specific markers for T-cells, B-cells, macrophages, and CAFs were used for analysis of the

immune and stroma infiltrate. Calculation of proliferation and stroma score: unique genes from merged G2M checkpoint and E2F targets pathways were selected based on absolute log2 fold change ≥1 (from DESeq2 analysis) between responders and non-responders. The product of this analysis constituted the proliferation score. Within the CAF signature, the top 10 genes with statistically significant upregulation in responding tumors (unadjusted p-value) constituted the stroma signature. The final proliferation and stroma scores were calculated by taking geometric means of respective gene sets. Immunofluorescence images were analysed by Imaris version 9 (Bitplane) and Fiji. Flow cytometry data was analysed by FlowJo software version 10 (Tree Star Inc.). Statistical tests were performed using GraphPad Prism version 7 and R.

For manuscripts utilizing custom algorithms or software that are central to the research but not yet described in published literature, software must be made available to editors/reviewers. We strongly encourage code deposition in a community repository (e.g. GitHub). See the Nature Research guidelines for submitting code & software for further information.

Data

Policy information about availability of data

All manuscripts must include a data availability statement. This statement should provide the following information, where applicable:

- Accession codes, unique identifiers, or web links for publicly available datasets
- A list of figures that have associated raw data
- A description of any restrictions on data availability

Sequencing data from mouse samples that support the findings of this study (Fig. 1-4 and Supplementary Fig. 2-4,6) have been deposited in the European Nucleotide Archive (ENA) with accession code PRJEB35895. Sequencing data from MDACC human cohort that support the findings of this study (Fig. 3-4 and Supplementary Fig. 5-6) have been deposited in the European Genome-phenome Archive (EGA) with accession code EGAS00001003178. All other relevant data are available from the corresponding author on reasonable request.

Field-specific reporting

Please select the one below that is the best fit for your research. If you are not sure, read the appropriate sections before making your selection.

☒ Life sciences ☐ Behavioural & social sciences ☐ Ecological, evolutionary & environmental sciences

For a reference copy of the document with all sections, see nature.com/documents/nr-reporting-summary-flat.pdf

Life sciences study design

All studies must disclose on these points even when the disclosure is negative.

Sample size	The treated mouse cohorts included a minimum of 13 animals which were deemed to be sufficient to provide statistically significant survival rates comparisons based on our historical precedent (Viros et al., Nature 2014; Trucco et al., Nature Med 2019). Human 'MDACC cohort' from Amaria et al., Nature Med 2018. Human 'Riaz cohort' from Riaz et al., Cell 2017.
Data exclusions	Mouse survival was analyzed in mice bearing single tumors starting on the day the treatment commenced until tumor volume reached 1500 mm3. Animals were censored when sacrifice during treatment was independent of tumor growth.<br>In all the analyses involving the human cohort from Riaz et al. Cell 2017, patient 3 was excluded in agreement with the authors' annotations.
Replication	Mouse experiments: UVR treatments followed standard operating protocols, and UVR machines were calibrated regularly to ensure the strength of UVR emission was consistent throughout experiments. Animals were continuously enrolled into the study that was performed across two different facilities, and data were pooled for all the analyses.
Randomization	All mouse experiments were performed based on standard operating protocols. Mice were randomized to the different treatment groups on a rolling recruitment basis when they reached the appropriate age/tumor size. Responding and non-responding tumors from anti-PD-1 treated animals were randomly selected for sequencing.
Blinding	Mouse experiments: Investigators involved in the assessment of lymphocytic infiltration by immunofluorescence or flow cytometry were blinded for treatment and response during the analyses of the samples. Pathologists evaluating mitotic index and Ki67 staining were blinded for response to treatment during the evaluations. Bioinformaticians evaluating the tumour mutational/neoantigens load were blinded for response to treatment during the analysis.

Reporting for specific materials, systems and methods

We require information from authors about some types of materials, experimental systems and methods used in many studies. Here, indicate whether each material, system or method listed is relevant to your study. If you are not sure if a list item applies to your research, read the appropriate section before selecting a response.

Materials & experimental systems

n/a	Involved in the study	
☐	☒	Antibodies
☒	☐	Eukaryotic cell lines
☒	☐	Palaeontology
☐	☒	Animals and other organisms
☐	☒	Human research participants
☒	☐	Clinical data

Methods

n/a	Involved in the study	
☒	☐	ChIP-seq
☐	☒	Flow cytometry
☒	☐	MRI-based neuroimaging

Antibodies

Antibodies used

In vivo treatment: anti-PD-1 (BioXcell; #BE0146; clone RMP1-14; 10 mg kg^-1 twice weekly by i.p. injection) or rat IgG2a (BioXcell; #BE0089; clone 2A3; 10 mg kg^-1 twice weekly by i.p. injection).
Flow cytometry: the following antibodies were purchased from BD Biosciences: anti-CD16/32 (BD Biosciences; #553142; clone 2.4G2; 1:1000), CD3-BUV737 (#564380; clone 17A2; 1:400), CD8a-BB515 (#564422; clone 53-6.7; 1:400), CD44-APC-Cy7 (#560568; clone IM7; 1:1000), CD274-BV711 (#563369; clone MIH5; 1:200), CD11b-FITC (#557672; clone M1/70; 1:800), CD45-BV605 (#563053; clone 30F11; 1:400). The following antibodies were purchased from Biolegend: CD4-BV510 (#100449; clone GK1.5; 1:400), CD62L-BV785 (#104440; clone MEL-14; 1:200), CD161-PE (#108707; clone PK136; 1:400), CD11c-PE (#117308; clone N418; 1:200), F4/80-AF647 (#123122; clone BM8; 1:2000).
Immunofluorescence: the following anti-mouse primary antibodies from Thermo Fisher Scientific were used: CD3-eFluor660 (#50-0032; clone 17A2, 1:200), CD4-eFluor570 (#41-0042; clone RM4-5; 1:50), and CD8a-eFluor615 (#42-0081; clone 53-6.7; 1:50). Anti-mouse PD-1 (clone RMP1-14; 7.2 mg ml^-1, 1:500) was produced in house by Louis Boon. This was detected by AlexaFluor488 conjugated anti-rat IgG secondary antibody (Stratech Scientific; #712-545-153; 1:400).
IHC: anti-Ki67 (Bethyl Laboratories; #IHC-00375) and secondary anti-rabbit (Dako; #K4003)

Validation

All antibodies are commercially available and were only used for applications validated by the manufacturer

Animals and other organisms

Policy information about studies involving animals; ARRIVE guidelines recommended for reporting animal research

Laboratory animals

Braf(+/LSL-V600E);Tyr::CreERT2(+/0) line was backcrossed over 10 generations to C57BL/6J. Mice were recruited onto experiments at 8 weeks of age. Only female mice were used.

Wild animals

The study did not involve wild animals

Field-collected samples

The study did not involve collection of samples from the field

Ethics oversight

All procedures involving animals were performed under the Home Office approved project license PE3DF1A5B, in accordance with ARRIVE guidelines and UK Home Office regulations under the Animals (Scientific Procedures) Act 1986. The study received ethical approval by the Cancer Research UK Manchester Institute’s Animal Welfare and Ethics Review Body (AWERB).

Note that full information on the approval of the study protocol must also be provided in the manuscript.

Human research participants

Policy information about studies involving human research participants

Population characteristics

Supplementary Figure 1a - CT scans from a single patient pre- and 12 weeks on-treatment with anti-PD-1 are included in the study. The patient was treated at the Christie Hospital (Manchester, UK) and response to therapy was assessed by RECIST 1.1 at 12 weeks of treatment.
Patient characteristics for the included RNAseq data are described for the MDACC cohort in Amaria et al., Nature Med 2018 and Helmink et al., Nature 2020. The second cohort is described in Riaz et al., Cell 2017.

Recruitment

Supplementary Figure 1a - The patient achieved mixed response to treatment.
Trial recruitment for the included RNAseq data is described for the MDACC cohort in Amaria et al., Nature Med 2018 and in Riaz et al., Cell 2017 for the second cohort of patients.

Ethics oversight

CT scans in Supplementary Figure 1a: Images from CT scan were obtained under the Manchester Cancer Research Centre (MCRC) Biobank ethics application #07/H1003/161+5 with full informed consent from the patient at The Christie NHS Foundation Trust. The work was approved by MCRC Biobank Access Committee application 13_RIMA_01. Response to treatment was assessed at 12-16 weeks by radiographic imaging using Response Evaluation Criteria In Solid Tumors, version 1.1 (RECIST v1.1).
MDACC patients: NCT02519322 trial was approved by the MD Anderson Cancer Center Institutional Review Board. The trial was conducted in accordance with the ethical principles of the Declaration of Helsinki and with adherence to the Good Clinical Practice guidelines, as defined by the International Conference on Harmonization. The protocol was conducted with compliance with all relevant ethical regulations and written informed consent was obtained from all participants.

Note that full information on the approval of the study protocol must also be provided in the manuscript.

Flow Cytometry

Plots

Confirm that:

☒ The axis labels state the marker and fluorochrome used (e.g. CD4-FITC).

☒ The axis scales are clearly visible. Include numbers along axes only for bottom left plot of group (a 'group' is an analysis of identical markers).

☒ All plots are contour plots with outliers or pseudocolor plots.

☒ A numerical value for number of cells or percentage (with statistics) is provided.

Methodology

Sample preparation	Tumors were minced and digested by using the mouse Tumor Dissociation Kit (Miltenyi Biotech), washed with FACS buffer (PBS containing 2% FBS, 2 mM EDTA and 0.02% sodium azide) and filtered through a 70 µm filter (BD Biosciences). The obtained single-cell suspension was stained with LIVE/DEAD Fixable Blue Dead Cell Stain kit (Thermo Fisher Scientific), blocked with anti-CD16/32 (BD Biosciences; #553142; clone 2.4G2; 1:1000), stained with fluorochrome labelled antibodies and analyzed.
Instrument	Samples were acquired by using a LSR Fortessa (BD Biosciences).
Software	Data was acquired by BD FACSDiva v7 (BD Biosciences) and analysed by FlowJo v10 (Tree Star Inc)
Cell population abundance	na
Gating strategy	Boundaries between positive and negative populations were set based on fluorescence minus one samples.

☒ Tick this box to confirm that a figure exemplifying the gating strategy is provided in the Supplementary Information.
