# Supplementary material for: Stroma remodeling and reduced cell division define durable response to PD-1 blockade in melanoma
Source: Nat Commun. 2020 Feb 12;11:853. doi: 10.1038/s41467-020-14632-2 (PMC7015935; doi:10.1038/s41467-020-14632-2)
Supplement: Supplementary file 1 — Peer Review File [file 41467_2020_14632_MOESM1_ESM.pdf]

Editorial Note: This manuscript has been previously reviewed at another journal that is not operating a transparent peer review scheme. This document only contains reviewer comments and rebuttal letters for versions considered at *Nature Communications*.

REVIEWERS' COMMENTS:

Reviewer #5 (Remarks to the Author):

The authors responded adequately to my prior, remaining critiques.

Response to Reviewer's comments:

Comment: *The authors responded adequately to my prior, remaining critiques.*

Response: We thank the Reviewer for their constructive comments which helped to improve the quality of our manuscript and we are delighted to note that we have adequately addressed all concerns leading to acceptance of our work.
